# Neuroimmune Interaction in Inflammatory Diseases

**DOI:** 10.4137/ccrpm.s547

**Published:** 2008-04-29

**Authors:** Peyman Otmishi, Joshiah Gordon, Seraj El-Oshar, Huafeng Li, Juan Guardiola, Mohamed Saad, Mary Proctor, Jerry Yu

**Affiliations:** Pulmonary and Critical Care, Department of Medicine, Ambulatory Care Building, 3rd floor University of Louisville, Louisville, KY 40292, U.S.A

**Keywords:** vagus nerve, sensory nerve, inflammation, immune, autonomic nervous system

## Abstract

The inflammatory response is modulated through interactions among the nervous, endocrine, and immune systems. Intercommunication between immune cells and the autonomic nervous system is a growing area of interest. Spatial and temporal information about inflammatory processes is relayed to the central nervous system (CNS) where neuroimmune modulation serves to control the extent and intensity of the inflammation. Over the past few decades, research has revealed various routes by which the nervous system and the immune system communicate. The CNS regulates the immune system via hormonal and neuronal pathways, including the sympathetic and parasympathetic nerves. The immune system signals the CNS through cytokines that act both centrally and peripherally. This review aims to introduce the concept of neuroimmune interaction and discuss its potential clinical application, in an attempt to broaden the awareness of this rapidly evolving area and open up new avenues that may aid in the treatment of inflammatory diseases.

## Neural Regulation of the Immune System

The CNS regulates the immune system through hormonal and neuronal pathways. The Hypothalamic-Pituitary-Adrenal (HPA) axis is the most important neuroendocrine pathway. Corticotropin releasing hormone (CRH) released from the paraventricular nucleus of the hypothalamus into the hypophyseal portal circulation causes the anterior pituitary gland to secrete adrenocorticotropin (ACTH). ACTH in turn induces the expression and release of glucocorticoids from the adrenal gland. Glucocorticoids suppress the immune system and have been used extensively in the treatment of inflammatory diseases since the 1940s (Webster, Tonelli, and Sternberg, 2002). In addition to affecting immune cell trafficking, migration, maturation and differentiation, glucocorticoids regulate the expression of cytokines, adhesion molecules, and chemotactic factors (Eskandari, Webster, and Sternberg, 2003). Glucocorticoids shift the cytokine pattern from pro-inflammatory [interleukin-1 (IL-1) and tumor necrosis factor-alpha (TNF-α)] to anti-inflammatory (IL-10) (Elenkov and Chrousos, 1999). Glucocorticoids act through a cytosolic glucocorticoid receptor that eventually translocates into the nucleus and binds to specific DNA sequences called glucocorticoid response elements to regulate gene transcription (Aranda and Pascual, 2001). Abnormalities in the signaling pathways can over-express or underexpress certain inflammatory mediators, leading to diseases such as asthma (Leung et al. 1997), inflammatory bowel disease (Honda et al. 2000), and rheumatoid arthritis (Derijk et al. 2001). Other Hypothalamic-Pituitary-target axes, such as the Hypothalamic-Pituitary-Gonadal axis, have also been implicated in the immune response (Berczi, 2001). At physiological concentrations, estrogen enhances the immune response whereas androgens suppress it (Cutolo and Wilder, 2000). For this reason, autoimmune and inflammatory diseases are more common in females than in males (Olsen and Kovacs, 1996).

Dendritic cells are antigen presenting cells. Their bone marrow-derived precursors reach most tissue via the bloodstream, becoming resident immature dendritic cells. These cells mature after activation and initiate the immune responses toward foreign proteins (Caux, Liu, and Banchereau, 1995). After capturing antigens, dendritic cells migrate to the T cell-dependent lymphoid organs via the afferent lymph. Their migration depends on the cell maturation and on the concentration of neuropeptides released by the sensory neurons at the site of inflammation (Dunzendorfer et al. 2001). Thus, stimulation of sensory nerve during inflammation may recruit antigen presenting cells to the inflammatory sites and therefore, propagate and control the degree of response.

**Figure 1.**
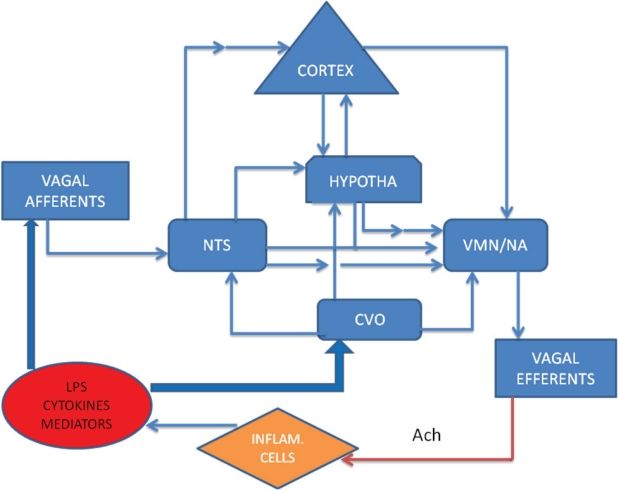
Illustration of a neuroimmune interaction. Inflammatory mediators and cytokines may activate vagal afferents, causing a ‘reflex’ via NTS to trigger neural circuitry and finally through vagal efferents to inhibit inflammatory cells to release inflammatory agents. The large, bold, blue arrows indicate initiation of the reflex pathway, while the thin, blue arrows denote a direct stimulatory effect; the red arrow indicates an inhibitory effect. Involvement of inter-neurons is indicated by two-arrow connections. LPS and high levels of cytokines and mediators may influence the higher brain centers via the CVO. **Abbreviations:** Ach: Acytylcholine; CVO: Circumventricular Organ; Hypotha: hypothalamus; Inflam. cells: inflammatory cells; NA: Nucleus Ambiguous; NTS: Nucleus Tractus Solitarus; LPS: Lipopolysacharide; VMN: Vagal Motor Nucleus.

Neural control of the immune system also occurs through the sympathetic and parasympathetic nerves. Both, in general, are anti-inflammatory. The sympathetic nerve innervates immune organs, such as the thymus, spleen and lymph nodes, causing effects through release of catecholamines. Immune cells (CD4+ T cells and B cells) express β_2_ adrenergic receptors, supporting direct regulation by neurotransmitters released from sympathetic efferents (Kin and Sanders, 2006). Catecholamines decrease pro-inflammatory cytokines, stimulate anti-inflammatory cytokines, and favor a shift from Th1 (cellular) to Th2 (humoral) responses (Elenkov and Chrousos, 1999). The parasympathetic nerves innervate most visceral organs and regulate the immune response through the so called ‘inflammatory reflex’ by a vagal-vagal mechanism (Tracey, 2002). In this scenario, vagal afferents are stimulated by pro-inflammatory cytokines (TNF-α and IL-1β) and mediators (histamine and prostaglandins) and reflexively activate vagal efferents, via the solitary nucleus, to release acetylcholine (Ach) (Fig. 1). Subsequently, Ach acts through alpha-nicotinic receptors on macrophages to inhibit production of pro-inflammatory cytokines (Borovikova et al. 2000; Wang et al. 2003a). Stimulation of vagal afferents by intraperitoneal injection of IL-1β produces sickness responses (fever, lethargy, etc.), which is blocked by sub-diaphragmatic vagotomy (Goehler et al. 1999). Vagal afferent activity is also increased by exogenous IL-1β injected into the receptive field of vagal sensory afferents in the lung (Yu et al. 2007) or by abdominal injection of lipopolysaccharides (LPS) that increase endogenous IL-1β. Electrical stimulation of vagal afferents increases IL-1β expression in the brain and activates HPA axis (Hosoi, Okuma, and Nomura, 2000). Electrical stimulation of vagal efferents during lethal endotoxemia inhibits TNF synthesis in the liver and lowers its serum level, preventing endotoxemic shock (Borovikova et al. 2000). Furthermore, using nicotine to activate the cholinergic anti-inflammatory pathways reduces mortality of endotoxemia (Wang et al. 2004) and inhibits endothelial cell activation and leukocyte migration (Saeed et al. 2005). Vagotomy nearly doubled mortality in septic animals (Kessler et al. 2006). Moreover, the discovery that mast cells interact with the nervous system through synaptic cell adhesion molecules (Ito and Oonuma, 2006) has shed light into the complex neuron-immune reaction. This vast undiscovered area of physiology has sparked interest and coined the term “the neuro-immune system” of disease.

## Influences of the Immune System on the CNS

The neuro-immune interaction is bidirectional through their soluble products: cytokines and mediators (Shepherd, Downing, and Miyan, 2005). Inflammatory cytokines play a crucial role in immune stimulation of nerves. Cytokines are a heterogenous group of polypeptides associated with activating the immune system and inflammatory responses. Cytokine receptors are found throughout the nervous system, including the CNS, vagus nerve, dorsal root ganglia, sciatic and sural nerves (Goehler et al. 1997; Plata-Salaman, 1991; Sorkin et al. 1997). IL-1β receptors are found even in carotid bodies (Wang et al. 2002). Activation of cytokine receptors contributes to neurological diseases such as multiple sclerosis, AIDS dementia complex, stroke, and Alzheimer’s disease (Benveniste, 1998; Hopkins and Rothwell, 1995; Rothwell and Hopkins, 1995). During acute inflammatory states the immune response includes systemic release of IL-1, which acts on the brain to activate the HPA axis to modulate the inflammatory response (Haddad, Saade, and Safieh-Garabedian, 2002).

Cytokines exert their effects on the CNS by various mechanisms, including: 1) by crossing the blood brain barrier through an active transport mechanism, 2) by passively diffusing through brain areas called circumventricular organs (CVO), where the fenestrated capillaries allow for higher permeability. Through the CVO, the brain can monitor levels of cytokines and mediators in the blood, and 3) by directly activating peripheral sensory nerves (Hosoi, Okuma, and Nomura, 2002). Pro-inflammatory cytokines or mediators may induce a series of mood and behavioral changes (collectively termed sickness behaviour, which is adaptive and helps the host to prevent system failure) by acting on receptors in the CNS, which are identical to those found on immune cells (Dantzer, 2004). The vagus nerve innervates a vast majority of internal organs, hence it can potentially receive various immune stimuli and convey immunological information to the CNS (Goehler et al. 1997; Watkins et al. 1995). In addition, fever develops within minutes following the peripheral injection of LPS when concentrations are not adequate to affect the brain in time to account for the response (Hosoi, Okuma, and Nomura, 2002; Kluger, 1991). These observations support a neural pathway that conveys peripheral immune signals to the brain and suggest a surveillance role of the CNS in numerous systemic diseases related to organ dysfunction like Irritable Bowel Syndrome and Acute Lung Injury.

In summary, the nervous system regulates the immune responses. Inflammation, a common mechanism involved in the pathophysiological process of various diseases, is an integral part of innate immune responses. Inflammatory products activate sensory nerves and send signals regarding the state of the inflammation to the CNS. Furthermore, through the reflex, efferent nerves can release mediators that act on immune cells (Fig. 1). In the following sections, we will discuss some particular inflammatory diseases associated with neuroimmune interaction.

## Rheumatology

Rheumatoid arthritis (RA) affects 1% of the general population, making it the most common form of chronic inflammatory arthritis. RA can be divided into two main phases: pre-clinical and clinical. The pre-clinical phase is asymptomatic (Vassilopoulos and Mantzoukis, 2006), when autoantibodies can be detected in blood (Nielen et al. 2004). Neuronendocrine interaction plays a crucial role at this stage. The clinical phase is symptomatic and can be further divided into early and chronic phases. Chronic inflammation alters neuropeptide processing, leading to long-term functional and structural changes in innervation, neurovascular regulation, and immune modulation. An altered HPA axis in RA points to an interaction between the disease and the neuroendocrine system.

The symmetric involvement of joints in RA and autonomic dysfunction associated with RA provides a compelling link of the disease with the CNS. Additionally, patients with central or peripheral hemiparesis who later develop RA, do not have joint disease in the paretic limbs, further suggesting neuronal involvement. In Sprague Dawley rats, inflammation (swelling and hyperalgesia) induced in one hindpaw produced the same signs in the contralateral paw with a similar magnitude and duration (Levine et al. 1985b). Chronic denervation of either the injured or the uninjured limb markedly attenuated the contralateral inflammatory response. This suggests that local inflammation can stimulate sensory nerves and reflexively produce inflammatory responses in the contralateral limb. In addition, denervation by capsaicin in either hind limb attenuates the contralateral responses, indicating an involvement of afferent C-fibers. Vein ligation has no effect on the inflammatory responses observed, excluding the possibility of humoral mediation (Levine et al. 1985a).

In physiological states, synovial nociceptors (C fibers and Aδ fibers) remain silent. During inflammation, however, they become sensitized to chemical, mechanical and thermal stimulation via two mechanisms. First, the mechanosensitive myelinated A fibers mediate sensitization at the pre-synaptic (CNS) level through repeated, prolonged stimulation. Second, soluble cytokines and inflammatory mediators (prostaglandins, adenosine, serotonin, and bradykinin) can directly sensitize neurons, including nociceptors (Niissalo et al. 2002). TNF-α and IL-1β play vital roles in joint inflammation and bone destruction. Immun-modulators such as steroids, anti-TNF, and anti-IL-1 have become the mainstay treatment in severe RA. The immune response is predominantly regional, but can be systemic. Substance-P (SP) released by sensory fibers in the inflamed synovium, cytokines such as TNF-α and IL-2, and circulating activated immune cells have all been implicated in rheumatoid pathology (Straub and Cutolo, 2001). For example, SP, which is pro-inflammatory, is secreted by nerves and inflammatory cells such as macrophages, eosinophils, lymphocytes and dendritic cells and acts through its neurokinin-1 (NK-1) receptor. SP and NK-1 receptor mRNA levels are elevated in the synovium and synoviocytes of patients with RA. Joint inflammation involves plasma extravasation, infiltration of the synovium with inflammatory cells, followed by vascular remodeling. SP potentiates plasma extravasation and enhances inflammatory responses in a mouse arthritis model (Keeble et al. 2005). Inflammatory signals activate the HPA axis and the hypothalamic-autonomic nervous system axis, exerting anti-inflammatory effects in arthritic joints through cortisol and catecholamines. In theory, an inadequate neuroendocrine response may result in the transition from the early to chronic phase in RA. Sympathetic responses are increased in RA patients following stimulation of sensory fibers in synovial tissue. Despite this, the number of sympathetic fibers in inflamed RA joints is lower than in non-inflammatory joints (Miller et al. 2000). Perhaps this loss of sympathetic fibers prevents the host from conveying anti-inflammatory actions through the sympathetic system.

Following exposure to streptococcal cell wall antigen, chronic arthritis develops in Lewis rats, which have a blunted HPA response, but not in Fischer rats having an increased hypothalamic CRH response. Glucocorticoid antagonists increase the susceptibility to arthritis, while glucocorticoids suppress the inflammatory response in arthritic rats (Vassilopoulos and Mantzoukis, 2006). However, arthritis in rat models is a polygenic disease where the impact of genetic variability on phenotypic expression accounts for about one-third of the cases. In human RA, genetic analysis for disease susceptibility has been performed around the world. Human Leukocyte Antigen (HLA)-related genes may be involved in predisposition to disease in about one-third of the cases, whereas non-HLA regions are responsible for remainders (Steinsson and arcon-Riquelme, 2005). Newly developed gene identification techniques may help determine if genes associated with the neuroendocrine axis play a role in RA predisposition.

The neuroendocrine link has also been implicated in Sjogren’s syndrome (Johnson and Moutsopoulos, 2000), chronic fatigue syndrome and fibromyalgia (Moldofsky, 1995). Sjogren’s syndrome is a perfect model for studying alterations in the neuroendocrineimmune system, since patients are usually medication free and hence devoid of the confounding effects of immunosuppressive treatment (Johnson and Moutsopoulos, 2000).

## Neurological Diseases

There has been much interest in the relationship between inflammation and pain. Painful information (nociception) is relayed to the CNS through afferent nerves, which are mostly comprised of unmyelinated C fibers and myelinated Aδ fibers. These afferents synapse to second order neurons in the dorsal horn of the spinal cord. Nociceptors are usually polymodal, responding to a variety of noxious stimuli (Kidd and Urban, 2001). Tissue inflammation or injury generates a surge of inflammatory mediators (cations, bradykinin, histamine, 5 HT, ATP, nitric oxide and cytokines). These mediators can either directly stimulate or sensitize the nociceptors. They can also increase production and release of more inflammatory mediators, leading to nociceptor activation. Hence, pro-inflammatory mediators may play a major role in the pathogenesis of chronic neuropathic pain syndromes.

Painful neuropathies are caused by nerve injury from inflammatory disease or trauma, and are influenced by cytokines and immune mediators (Baron, 2000). Their study can be traced back to Mitchell’s classic work from the American Civil War (Moalem and Tracey, 2006). Nerve injury activates resident immune cells and recruits inflammatory cells to the injured sites, initiating an inflammatory cascade. Mast cells near the nerve are activated to release histamine and TNF-α, which recruit neutrophils and macrophages, as well as sensitize nociceptors. In turn, the recruited inflammatory cells secrete cytokines and mediators (TNF-α and PGE_2_) that can further sensitize nociceptors. Nerve injury releases mediators, including ATP, nerve growth factor, PGE_2_ and pro-inflammatory cytokines. This promotes recruitment of T cells, secreting a variety of cytokines. This cocktail of mediators enhances the inflammation and contributes to pain (Moalem and Tracey, 2006). Focal neuritis in the rat sciatic nerve produces neuropathic pain sensations. Neuroimmune interaction occurs at the outset of nerve injury that produces neuropathic pain. For example, TNF-α can be released from sciatic Schwann cells (Wagner and Myers, 1996) and injection of TNF-α and IL-1β into rat sciatic nerve causes pain (Zelenka, Schafers, and Sommer, 2005). TNF-α can stimulate the sciatic nerve directly. Intradermal injection of TNF-α leads to sensitization and subsequent hyperalgesia (Sorkin et al. 1997). Such an interaction may prime the system for slowly developing sustained pain during the chronic phase of neuropathy (Bennett, 1999). Over time, episodic priming, by the release of inflammatory mediators, may also contribute to chronic conditions like diabetic neuropathy. Guillain-Barré Syndrome, an autoimmune injury of the peripheral nervous system causing demyelination, is characterized by progressive ascending polyneuropathy, which may progress to quadriplegia and lead to respiratory arrest. A strong correlation of serum levels of TNF-α and its receptors with disease severity and recovery is found in patients with Guillain-Barré Syndrome (Radhakrishnan et al. 2003).

Sympathetic nerve has been investigated in various studies related to neuroimmune interaction, including the pathogenesis of multiple sclerosis (Elenkov et al. 2000). Receptors for sympathetic neural transmitter, β2 adrenoceptors, provide important protective and supportive functions in cell proliferation, response, and metabolism. Astrocytes in multiple sclerosis lack β2 adrenoceptors (De et al. 2004), the activation of which inhibits the expression of pro-inflammatory cytokines via cAMP. Lack of β2 adrenoceptors may also transform astrocytes into facultative antigen presenting cells, initiating the inflammatory cascade. β2 receptor defects also impair glycogenolysis, which generates lactate as an energy source for axons. Disturbances in axonal metabolism may cause accumulation of calcium ions inside the axon and thus degeneration. This rationale might provide a new therapeutic strategy to reduce or prevent both relapses and the progression of multiple sclerosis by increasing c-AMP levels in astrocytes (De et al. 2004).

## Gastrointestinal Tract

Recent research demonstrates the interrelationship between the nervous system and the mucosal immune system (Shanahan, 1999). In the gastrointestinal (GI) tract, activated mast cells release inflammatory mediators, which in turn stimulate sensory nerve endings (De Jonge et al. 2005). Activation of sensory nerves initiates ‘the inflammatory reflex’, releasing acetylcholine from vagal efferents and inhibiting cytokine production by macrophages (De Jonge et al. 2005). Furthermore, vagotomy leads to increase levels of IL-6, TNF-α and IL-1β in the gut (Ghia et al. 2006), exemplifying the vagal anti-inflammatory effects. Such a mechanism explains a well-established relationship between tobacco use (nicotine) and inflammatory bowel disease (IBD). In ulcerative colitis, the protective effect of nicotine is related to the α7-subunit and its inhibition of TNF-α (Johnson, Cosnes, and Mansfield, 2005) and IL-8 (Louvet et al. 1999). Bowel manipulation, transplantation, and ischemia/reperfusion injury activate intestinal macrophages and cause acute gut inflammation. The pathogenesis of post-operative ileus exemplifies such a mechanism. Macrophages are activated in the muscularis externa of the human small bowel following intestinal manipulation (Kalff et al. 2003). Once activated, these resident macrophages release pro-inflammatory mediators that cause bowel inflammation and inhibit contractility. Local inflammation can also inhibit distant sites, demonstrating that a neural pathway can paralyze the entire GI tract (de Jonge et al. 2003).

Inflammatory bowel disease, including Crohn’s disease and ulcerative colitis, is characterized by overproduction of inflammatory cytokines, dysregulation of mucosal immunity and uncontrolled inflammation in the gut. In patients with IBD, structural abnormalities include enteric glial cell hyperplasia (Geboes and Collins, 1998), ganglion cell and axonal degeneration, and necrosis (Dvorak et al. 1993). Functionally, enteric glial cells are equivalent to astrocytes in the CNS (Cabarrocas, Savidge, and Liblau, 2003). Enteric glial cells and neurons in Crohn’s Disease express a major histocompatibility complex class 2 antigen on their cell surface (similar to macrophages) allowing them to act as antigen presenting cells and release pro-inflammatory cytokines and mediators (Geboes et al. 1992). SP has been implicated in IBD pathology. In IBD mouse models, NK-1 receptors (the binding sites for SP) were up-regulated in intestinal tissue. NK-1 receptor antagonists decreased disease severity and promoted healing pre-existing lesions (Sonea et al. 2002). Infliximab, an antibody, which neutralizes TNF-α and down-regulates interferon gamma production (Agnholt and Kaltoft, 2001), benefits patients with active steroid-dependent or with fistulizing Crohn’s disease. All stated above are consistent with that neuroimmune interactions play a role in the pathogenesis of IBD. Perhaps new avenues will open up for designing drugs to modify the disease course and decrease the morbidity of IBD.

Irritable bowel syndrome (IBS) is the consequence of altered visceral perception and regulation of gastrointestinal motility and epithelial function, and is associated with CNS disturbance and psycosocial stresses (Barbara et al. 2004). The syndrome may accrue from primary alterations in the periphery or in the CNS, or a combination (Mayer, Naliboff, and Chang, 2001). Brain-gut interactions are mediated by outputs from the autonomic and neuroendocrine systems, as well as from attentional and pain modulatory responses. Psychological and physical stressors affect function of the GI tract through neuronal activity within emotion-specific circuits in the brain, and therefore enhancing gastric motility, mucosal blood flow and acid secretion. In response to internal or external stress, pro-inflammatory cytokines in the GI tract are increased. This causes perception of symptoms via intrinsic and extrinsic primary afferent neurons (Barbara et al. 2004), which in turn further activates the neuronal circuits, intensifying the symptoms. Thus, IBS patients have an enhanced GI responsiveness due to malfunction of the neuroendocrine-immune interaction (Mulak and Bonaz, 2004). In the last 20 years, low dose antidepressants have been successfully used to treat IBS patients, partly due to their neuromodulator properties, in addition to their analgesic effects (Wald, 2002).

Lastly, neuroimmune interaction may also account for some cases of appendicitis that are negative for inflammation on histopathology. These are associated with neuroproliferation and increased SP and vasointestinal polypeptides. This distinct pathological entity, neuroimmune appendicitis, explains up to 25% of normal appendixes in surgical patients (Di et al. 1999). In experimental pancreatitis, the vagus nerve provides a protective function through anti-inflammatory effects via nicotinic receptors (van Westerloo et al. 2006). This may result in new approaches to treating acute pancreatitis besides bowel rest and pain analgesia.

## Respiratory System

Pulmonary nociceptors, the most abundant afferent nerves in the lung, are sensitive to inflammatory products, such as histamine, prostaglandins, and SP (Coleridge and Coleridge, 1984; Lee and Pisarri, 2001). Rodent nociceptors are associated with neuropeptides, SP, tachykinins, and Calcitonin Gene-Related Peptide, as well as other neurokinins. These neuropeptides are responsible for neurogenic inflammation through the interaction of nociceptive fibers and alveolar macrophages, releasing neuropeptides and cytokines, respectively (McDonald, 1987; Solway and Leff, 1991). TNF-α increases bronchial tissue responsiveness (Anticevich et al. 1995), suggesting a role of inflammatory cytokines in asthma and COPD. Accumulating evidence indicates the neuroendocrine and immune systems interact during the development of asthma (Marshall, Jr. and Agarwal, 2000). Many mediators released in the inflammatory zone may modulate sensory and motor nerves in the airways (Barnes, 1992). Sensory nerves, in turn, may amplify inflammation through the release of peptide neurotransmitters. Electrical stimulation of the cervical vagus nerve evokes neurogenic inflammation in the trachea and bronchi of rats. Neural mediators modulate the pulmonary inflammatory response by triggering vasodilatation, tissue edema, and influx of inflammatory cells (Kraneveld and Nijkamp, 2001). SP and neurokinin are involved in bronchoconstriction and mucous production. The presence of SP in sensory fibers is the groundwork of neurogenic inflammation. Interestingly, mechanosensors in the lung, which do not express SP, become SP immune reactive after allergen challenge in guinea pigs. Ipsilateral vagotomy suppresses the SP expression (Chuaychoo et al. 2005). Nerve growth factor, which is closely associated with asthma, is released from lung fibroblasts at an increased rate when exposed to TNF-α and IL-1β (Olgart and Frossard, 2001). Pulmonary afferents, traveling through sympathetic nerves, also exist (Soukhova et al. 2003; Wang et al. 2003b) and potentially contribute to lung pathophysiology. Acute hypoxia influences the neuroimmune system through the action of IL-1β under numerous conditions (asthma, chronic obstructive lung disease, sleep apnea and heart failure) (Johnson et al. 2007). In addition, emotions have a clear influence on asthmatic attack. A number of studies have found a relationship between asthma and negative emotions, such as anxiety, anger, and sadness (von, Ehnes, and Dahme, 2006), supporting a neuroimmune interaction.

Acute Respiratory Distress Syndrome (ARDS) is a major source of intensive care mortality because of its association with other acute disorders (infection, trauma, sepsis, intoxication, or pancreatitis). Pro-inflammatory cytokines may be crucial in the initiation, propagation, maintenance and regression of the inflammatory response in the lungs by stimulating pulmonary nociceptors. Pro-inflammatory cytokines are markedly elevated in broncho-alveolar lavage (BAL) samples from patients with ARDS, or those at risk for ARDS (Siler et al. 1989; Suter et al. 1992). TNFα, IL-1β and IL- 6 in plasma and BAL are increased in non-survivors of ARDS (Meduri et al. 1995). Furthermore, pulmonary nociceptor activity is increased dramatically during oleic acid-induced ARDS (Lin et al. 2007) or by direct injection of the inflammatory cytokine IL-1β into the sensory receptive field (Yu et al. 2007). This supports neuroimmune interaction mechanisms during acute pulmonary events.

## Conclusion

As a mechanism, neuroimmune interaction controls inflammation in multiple diseases. The connection between the brain and inflammatory diseases is not restricted to the previous examples. Brain-skin interaction is increasingly recognized. For example, atopic dermatitis patients demonstrate a blunted HPA axis response (Buske-Kirschbaum et al. 2002). Neurokinins derived from sensory nerves interact with antigen presentation in dermal Langerhans cells in allergic skin disease (Darsow and Ring, 2001). Intracerebral administion of melanocyte-stimulating hormone α can inhibit cytokine-induced cutaneous inflammation (Brazzini et al. 2003). Inflammation also is a crucial component in cardiovascular diseases, including atherosclerosis. The autonomic nervous system may perform heart-brain communication through cytokine signaling to alter the disease course through anti-inflammatory actions (Shishehbor, Alves, and Rajagopal, 2007). Neuropsychiatric disorders are influenced by neuroimmune interaction via neurotransmitters and cytokines (Fricchione et al. 2001). Neuroendocrine-immune cross-talk is not limited to inflammation as a triggering factor. Alcohol, for example, may serve as a neurochemical regulator of the HPA axis (Haddad, 2004). Understanding this interaction should facilitate treatment of alcoholic addiction.

In short, research has advanced our understanding of neuroimmune interaction in disease processes over the last decade. This new understanding has introduced a new approach to a variety of disease processes. Both pro-inflammatory cytokines and immune cells have been targeted for the treatment of various diseases. With intensified research, detailed underlying mechanisms will be revealed and innovative strategies will be developed to alter the course of currently debilitating and life threatening diseases.

## References

[b1-ccrpm-2008-035] Agnholt J Kaltoft K 2001 Infliximab downregulates interferon-gamma production in activated gut T-lymphocytes from patients with Crohn’s disease Cytokine 15 4 212 22 11563881 10.1006/cyto.2001.0919

[b2-ccrpm-2008-035] Anticevich SZ Hughes JM Black JL Armour CL 1995 Induction of human airway hyperresponsiveness by tumour necrosis factor-alpha Eur. J. Pharmacol 284 1–2 221 5 8549631 10.1016/0014-2999(95)00463-u

[b3-ccrpm-2008-035] Aranda A Pascual A 2001 Nuclear hormone receptors and gene expression Physiol. Rev 81 3 1269 304 11427696 10.1152/physrev.2001.81.3.1269

[b4-ccrpm-2008-035] Barbara G De GR Stanghellini V Cremon C Salvioli B Corinaldesi R 2004 New pathophysiological mechanisms in irritable bowel syndrome Aliment. Pharmacol. Ther 20 Suppl 2 1 9 15335408 10.1111/j.1365-2036.2004.02036.x

[b5-ccrpm-2008-035] Barnes PJ 1992 Modulation of neurotransmission in airways Physiol. Rev 72 3 699 729 1352637 10.1152/physrev.1992.72.3.699

[b6-ccrpm-2008-035] Baron R 2000 Peripheral neuropathic pain: from mechanisms to symptoms Clin. J. Pain 16 2 S12 S20 10870735 10.1097/00002508-200006001-00004

[b7-ccrpm-2008-035] Bennett GJ 1999 Does a neuroimmune interaction contribute to the genesis of painful peripheral neuropathies? Proc. Natl. Acad. Sci. U.S.A 96 14 7737 8 10393890 10.1073/pnas.96.14.7737 PMC33611

[b8-ccrpm-2008-035] Benveniste EN 1998 Cytokine actions in the central nervous system Cytokine Growth Factor Rev 9 3–4 259 75 9918124 10.1016/s1359-6101(98)00015-x

[b9-ccrpm-2008-035] Berczi I 2001 Neuroimmune Biology—An Introduction Neuroimmune Biology: New Foundation of Biology Berczi I Gorczynski R M Elsevier B.V., Shannon, Co Clare, Ireland 3 45

[b10-ccrpm-2008-035] Borovikova LV Ivanova S Zhang M Yang H Botchkina GI Watkins LR Wang H Abumrad N Eaton JW Tracey KJ 2000 Vagus nerve stimulation attenuates the systemic inflammatory response to endotoxin Nature 405 6785 458 62 10839541 10.1038/35013070

[b11-ccrpm-2008-035] Brazzini B Ghersetich I Hercogova J Lotti T 2003 The neuroimmuno-cutaneous-endocrine network: relationship between mind and skin Dermatol. Ther 16 2 123 31 12919114 10.1046/j.1529-8019.2003.01621.x

[b12-ccrpm-2008-035] Buske-Kirschbaum A Geiben A Hollig H Morschhauser E Hellhammer D 2002 Altered responsiveness of the hypothalamus-pituitary-adrenal axis and the sympathetic adrenomedullary system to stress in patients with atopic dermatitis J. Clin. Endocrinol. Metab 87 9 4245 51 12213879 10.1210/jc.2001-010872

[b13-ccrpm-2008-035] Cabarrocas J Savidge TC Liblau RS 2003 Role of enteric glial cells in inflammatory bowel disease Glia 41 1 81 93 12465048 10.1002/glia.10169

[b14-ccrpm-2008-035] Caux C Liu YJ Banchereau J 1995 Recent advances in the study of dendritic cells and follicular dendritic cells Immunol 16 1 2 4 10.1016/0167-5699(95)80061-1 7880384

[b15-ccrpm-2008-035] Chuaychoo B Hunter DD Myers AC Kollarik M Undem BJ 2005 Allergen-induced substance P synthesis in large-diameter sensory neurons innervating the lungs J. Allergy Clin. Immunol 116 2 325 31 16083787 10.1016/j.jaci.2005.04.005

[b16-ccrpm-2008-035] Coleridge JCG Coleridge HM 1984 Afferent vagal C fibre innervation of the lungs and airways and its functional significance Rev. Physiol. Biochem. Pharmacol 99 1 110 6695127 10.1007/BFb0027715

[b17-ccrpm-2008-035] Cutolo M Wilder RL 2000 Different roles for androgens and estrogens in the susceptibility to autoimmune rheumatic diseases Rheum. Dis. Clin. North Am 26 4 825 39 11084946 10.1016/s0889-857x(05)70171-x

[b18-ccrpm-2008-035] Dantzer R 2004 Cytokine-induced sickness behaviour: a neuroimmune response to activation of innate immunity Eur. J. Pharmacol 500 1–3 399 411 15464048 10.1016/j.ejphar.2004.07.040

[b19-ccrpm-2008-035] Darsow U Ring J 2001 Neuroimmune interactions in the skin Curr. Opin. Allergy Clin. Immunol 1 5 435 9 11964724 10.1097/01.all.0000011057.09816.61

[b20-ccrpm-2008-035] De Jonge WJ The FO van der Zanden EP van den Wijngaard RM Boeckxstaens GE 2005 Inflammation and gut motility; neural control of intestinal immune cell activation J. Pediatr. Gastroenterol. Nutr 41 Suppl 1 S10 S11 16131950 10.1097/01.scs.0000180287.58988.86

[b21-ccrpm-2008-035] de Jonge WJ van den Wijngaard RM The FO ter Beek ML Bennink RJ Tytgat GN Buijs RM Reitsma PH van Deventer SJ Boeckxstaens GE 2003 Postoperative ileus is maintained by intestinal immune infiltrates that activate inhibitory neural pathways in mice Gastroenterology 125 4 1137 47 14517797 10.1016/s0016-5085(03)01197-1

[b22-ccrpm-2008-035] De KJ Zeinstra E Mostert J Wilczak N 2004 Beta 2-adrenoceptor involvement in inflammatory demyelination and axonal degeneration in multiple sclerosis Trends Pharmacol.. Sci 25 2 67 71 15102491 10.1016/j.tips.2003.12.002

[b23-ccrpm-2008-035] Derijk RH Schaaf MJ Turner G Datson NA Vreugdenhil E Cidlowski J de Kloet ER Emery P Sternberg EM tera-Wadleigh SD 2001 A human glucocorticoid receptor gene variant that increases the stability of the glucocorticoid receptor beta-isoform mRNA is associated with rheumatoid arthritis J. Rheumatol 28 11 2383 8 11708406

[b24-ccrpm-2008-035] Di SP Fink T di Mola FF Weihe E Innocenti P Friess H Buchler MW 1999 Neuroimmune appendicitis Lancet 354 9177 461 6 10465170 10.1016/S0140-6736(98)10463-4

[b25-ccrpm-2008-035] Dunzendorfer S Kaser A Meierhofer C Tilg H Wiedermann CJ 2001 Cutting edge: peripheral neuropeptides attract immature and arrest mature blood-derived dendritic cells J. Immunol 166 4 2167 72 11160268 10.4049/jimmunol.166.4.2167

[b26-ccrpm-2008-035] Dvorak AM Onderdonk AB McLeod RS Monahan-Earley RA Cullen J Antonioli DA Blair JE Morgan ES Cisneros RL Estrella P 1993 Axonal necrosis of enteric autonomic nerves in continent ileal pouches. Possible implications for pathogenesis of Crohn’s disease Ann. Surg 217 3 260 71 8383954 10.1097/00000658-199303000-00008 PMC1242779

[b27-ccrpm-2008-035] Elenkov IJ Chrousos GP 1999 Stress Hormones, Th1/Th2 patterns, Pro/Anti-inflammatory Cytokines and Susceptibility to Disease Trends Endocrinol. Metab 10 9 359 68 10511695 10.1016/s1043-2760(99)00188-5

[b28-ccrpm-2008-035] Elenkov IJ Wilder RL Chrousos GP Vizi ES 2000 The sympathetic nerve—an integrative interface between two super-systems: the brain and the immune system Pharmacol. Rev 52 4 595 638 11121511

[b29-ccrpm-2008-035] Eskandari F Webster JI Sternberg EM 2003 Neural immune pathways and their connection to inflammatory diseases Arthritis Res.Ther 5 6 251 65 14680500 10.1186/ar1002 PMC333413

[b30-ccrpm-2008-035] Fricchione G Daly R Rogers MP Stefano GB 2001 Neuroimmunologic influences in neuropsychiatric and psychophysiologic disorders Acta. Pharmacol. Sin 22 7 577 87 11749820

[b31-ccrpm-2008-035] Geboes K Collins S 1998 Structural abnormalities of the nervous system in Crohn’s disease and ulcerative colitis Neurogastroenterol. Motil 10 3 189 202 9659662 10.1046/j.1365-2982.1998.00102.x

[b32-ccrpm-2008-035] Geboes K Rutgeerts P Ectors N Mebis J Penninckx F Vantrappen G Desmet VJ 1992 Major histocompatibility class II expression on the small intestinal nervous system in Crohn's disease Gastroenterology 103 2 439 47 1378803 10.1016/0016-5085(92)90832-j

[b33-ccrpm-2008-035] Ghia JE Blennerhassett P Kumar-Ondiveeran H Verdu EF Collins SM 2006 The vagus nerve: a tonic inhibitory influence associated with inflammatory bowel disease in a murine model Gastroenterology 131 4 1122 30 17030182 10.1053/j.gastro.2006.08.016

[b34-ccrpm-2008-035] Goehler LE Gaykema RP Nguyen KT Lee JE Tilders FJ Maier SF Watkins LR 1999 Interleukin-1beta in immune cells of the abdominal vagus nerve: a link between the immune and nervous systems? J. Neurosci 19 7 2799 806 10087091 10.1523/JNEUROSCI.19-07-02799.1999 PMC6786076

[b35-ccrpm-2008-035] Goehler LE Relton JK Dripps D Kiechle R Tartaglia N Maier SF Watkins LR 1997 Vagal paraganglia bind biotinylated interleukin-1 receptor antagonist: a possible mechanism for immune-to-brain communication Brain Res. Bull 43 3 357 64 9227848 10.1016/s0361-9230(97)00020-8

[b36-ccrpm-2008-035] Haddad JJ 2004 Alcoholism and neuro-immune-endocrine interactions: physiochemical aspects Biochem. Biophys. Res. Commun 323 2 361 71 15369760 10.1016/j.bbrc.2004.08.119

[b37-ccrpm-2008-035] Haddad JJ Saade NE Safieh-Garabedian B 2002 Cytokines and neuro-immune-endocrine interactions: a role for the hypothalamic-pituitary-adrenal revolving axis J. Neuroimmunol 133 1–2 1 19 12446003 10.1016/s0165-5728(02)00357-0

[b38-ccrpm-2008-035] Honda M Orii F Ayabe T Imai S Ashida T Obara T Kohgo Y 2000 Expression of glucocorticoid receptor beta in lymphocytes of patients with glucocorticoid-resistant ulcerative colitis Gastroenterology 118 5 859 66 10784585 10.1016/s0016-5085(00)70172-7

[b39-ccrpm-2008-035] Hopkins SJ Rothwell NJ 1995 Cytokines and the nervous system. I: Expression and recognition Trends Neurosci 18 2 83 8 7537419

[b40-ccrpm-2008-035] Hosoi T Okuma Y Nomura Y 2000 Electrical stimulation of afferent vagus nerve induces IL-1beta expression in the brain and activates HPA axis Am. J. Physiol. Regul. Integr. Comp. Physiol 279 1 R141 R147 10896875 10.1152/ajpregu.2000.279.1.R141

[b41-ccrpm-2008-035] Hosoi T Okuma Y Nomura Y 2002 The mechanisms of immune-to-brain communication in inflammation as a drug target Curr. Drug Targets Inflamm. Allergy 1 3 257 62 14561190 10.2174/1568010023344599

[b42-ccrpm-2008-035] Ito A Oonuma J 2006 Direct interaction between nerves and mast cells mediated by the SgIGSF/SynCAM adhesion molecule J. Pharmacol. Sci 102 1 1 5 16936456 10.1254/jphs.cpj06014x

[b43-ccrpm-2008-035] Johnson DR O’Connor JC Hartman ME Tapping RI Freund GG 2007 Acute hypoxia activates the neuroimmune system, which diabetes exacerbates J. Neurosci 27 5 1161 6 17267571 10.1523/JNEUROSCI.4560-06.2007 PMC6673177

[b44-ccrpm-2008-035] Johnson EO Moutsopoulos HM 2000 Neuroendocrine manifestations in Sjogren’s syndrome. Relation to the neurobiology of stress Ann. N.Y. Acad. Sci 917 797 808 11268409 10.1111/j.1749-6632.2000.tb05445.x

[b45-ccrpm-2008-035] Johnson GJ Cosnes J Mansfield JC 2005 Review article: smoking cessation as primary therapy to modify the course of Crohn’s disease Aliment. Pharmacol. Ther 21 8 921 31 15813828 10.1111/j.1365-2036.2005.02424.x

[b46-ccrpm-2008-035] Kalff JC Turler A Schwarz NT Schraut WH Lee KK Tweardy DJ Billiar TR Simmons RL Bauer AJ 2003 Intra-abdominal activation of a local inflammatory response within the human muscularis externa during laparotomy Ann. Surg 237 3 301 15 12616113 10.1097/01.SLA.0000055742.79045.7E PMC1514322

[b47-ccrpm-2008-035] Keeble J Blades M Pitzalis C Castro da Rocha FA Brain SD 2005 The role of substance P in microvascular responses in murine joint inflammation 10.1038/sj.bjp.0706131 PMC1576088 15700029

[b48-ccrpm-2008-035] Kessler W Traeger T Westerholt A Neher F Mikulcak M Muller A Maier S Heidecke CD 2006 The vagal nerve as a link between the nervous and immune system in the instance of polymicrobial sepsis Langenbecks Arch. Surg 391 2 83 7 16568325 10.1007/s00423-006-0031-y

[b49-ccrpm-2008-035] Kidd BL Urban LA 2001 Mechanisms of inflammatory pain Br. J. Anaesth 87 1 3 11 11460811 10.1093/bja/87.1.3

[b50-ccrpm-2008-035] Kin NW Sanders VM 2006 It takes nerve to tell T and B cells what to do J. Leukoc. Biol 79 6 1093 104 16531560 10.1189/jlb.1105625

[b51-ccrpm-2008-035] Kluger MJ 1991 Fever: role of pyrogens and cryogens Physiol. Rev 71 1 93 127 1986393 10.1152/physrev.1991.71.1.93 PMC7191625

[b52-ccrpm-2008-035] Kraneveld AD Nijkamp FP 2001 Tachykinins and neuro-immune interactions in asthma Int. Immunopharmacol 1 9–10 1629 50 11562057 10.1016/s1567-5769(01)00099-6

[b53-ccrpm-2008-035] Lee LY Pisarri TE 2001 Afferent properties and reflex functions of bronchopulmonary C-fibers Respir. Physiol 125 1–2 47 65 11240152 10.1016/s0034-5687(00)00204-8

[b54-ccrpm-2008-035] Leung DY Hamid Q Vottero A Szefler SJ Surs W Minshall E Chrousos GP Klemm DJ 1997 Association of glucocorticoid insensitivity with increased expression of glucocorticoid receptor beta J. Exp. Med 186 9 1567 74 9348314 10.1084/jem.186.9.1567 PMC2199113

[b55-ccrpm-2008-035] Levine JD Collier DH Basbaum AI Moskowitz MA Helms CA 1985a Hypothesis: the nervous system may contribute to the pathophysiology of rheumatoid arthritis J. Rheumatol 12 3 406 11 2413204

[b56-ccrpm-2008-035] Levine JD Dardick SJ Basbaum AI Scipio E 1985b Reflex neurogenic inflammation. I. Contribution of the peripheral nervous system to spatially remote inflammatory responses that follow injury J. Neurosci 5 5 1380 6 3998827 10.1523/JNEUROSCI.05-05-01380.1985 PMC6565043

[b57-ccrpm-2008-035] Lin S Walker J Xu L Gozal D Yu J 2007 Respiratory: Behaviours of pulmonary sensory receptors during development of acute lung injury in the rabbit Exp. Physiol 92 4 749 55 17392336 10.1113/expphysiol.2006.036673

[b58-ccrpm-2008-035] Louvet B Buisine MP Desreumaux P Tremaine WJ Aubert JP Porchet N Capron M Cortot A Colombel JF Sandborn WJ 1999 Transdermal nicotine decreases mucosal IL-8 expression but has no effect on mucin gene expression in ulcerative colitis Inflamm. Bowel. Dis 5 3 174 81 10453373 10.1097/00054725-199908000-00005

[b59-ccrpm-2008-035] Marshall GD Agarwal SK 2000 Stress, immune regulation, and immunity: applications for asthma Allergy Asthma Proc 21 4 241 6 10951892 10.2500/108854100778248917

[b60-ccrpm-2008-035] Mayer EA Naliboff BD Chang L 2001 Basic pathophysiologic mechanisms in irritable bowel syndrome Dig. Dis 19 3 212 8 11752839 10.1159/000050682

[b61-ccrpm-2008-035] McDonald DM 1987 Neurogenic inflammation in the respiratory tract: actions of sensory nerve mediators on blood vessels and epithelium of the airway mucosa Am. Rev. Respir. Dis 136 6 Pt 2 S65 S72 3318605 10.1164/ajrccm/136.6_Pt_2.S65

[b62-ccrpm-2008-035] Meduri GU Kohler G Headley S Tolley E Stentz F Postlethwaite A 1995 Inflammatory cytokines in the BAL of patients with ARDS Persistent elevation over time predicts poor outcome Chest 108 5 1303 14 7587434 10.1378/chest.108.5.1303

[b63-ccrpm-2008-035] Miller LE Justen HP Scholmerich J Straub RH 2000 The loss of sympathetic nerve fibers in the synovial tissue of patients with rheumatoid arthritis is accompanied by increased norepinephrine release from synovial macrophages FASEB J 14 13 2097 107 11023994 10.1096/fj.99-1082com

[b64-ccrpm-2008-035] Moalem G Tracey DJ 2006 Immune. and inflammatory mechanisms in neuropathic pain Brain Res. Rev 51 2 240 64 16388853 10.1016/j.brainresrev.2005.11.004

[b65-ccrpm-2008-035] Moldofsky H 1995 Sleep, neuroimmune and neuroendocrine functions in fibromyalgia and chronic fatigue syndrome Adv. Neuroimmunol 5 1 39 56 7795892 10.1016/0960-5428(94)00048-s

[b66-ccrpm-2008-035] Mulak A Bonaz B 2004 Irritable bowel syndrome: a model of the brain-gut interactions Med. Sci. Monit. 10 4 RA55 RA62 15260348

[b67-ccrpm-2008-035] Nielen MM van SD Reesink HW van de Stadt RJ van der Horst-Bruinsma IE de Koning MH Habibuw MR Vandenbroucke JP Dijkmans BA 2004 Specific autoantibodies precede the symptoms of rheumatoid arthritis: a study of serial measurements in blood donors Arthritis Rheum 50 2 380 6 14872479 10.1002/art.20018

[b68-ccrpm-2008-035] Niissalo S Hukkanen M Imai S Tornwall J Konttinen YT 2002 Neuropeptides in experimental and degenerative arthritis Ann. N.Y. Acad. Sci 966 384 99 12114296 10.1111/j.1749-6632.2002.tb04239.x

[b69-ccrpm-2008-035] Olgart C Frossard N 2001 Human lung fibroblasts secrete nerve growth factor: effect of inflammatory cytokines and glucocorticoids Eur. Respir. J 18 1 115 21 11510781 10.1183/09031936.01.00069901

[b70-ccrpm-2008-035] Olsen NJ Kovacs WJ 1996 Gonadal steroids and immunity Endocr. Rev 17 4 369 84 8854050 10.1210/edrv-17-4-369

[b71-ccrpm-2008-035] Plata-Salaman CR 1991 Immunoregulators in the nervous system Neurosci. Biobehav. Rev 15 2 185 215 1852312 10.1016/s0149-7634(05)80001-6

[b72-ccrpm-2008-035] Radhakrishnan VV Sumi MG Reuben S Mathai A Nair MD 2003Circulating tumour necrosis factor alpha and soluble TNF receptors in patients with Guillain-Barre syndrome. 14609050

[b73-ccrpm-2008-035] Rothwell NJ Hopkins SJ 1995 Cytokines and the nervous system II: Actions and mechanisms of action Trends Neurosci 18 3 130 6 7754524 10.1016/0166-2236(95)93890-a

[b74-ccrpm-2008-035] Saeed RW Varma S Peng-Nemeroff T Sherry B Balakhaneh D Huston J Tracey KJ Al-Abed Y Metz CN 2005 Cholinergic stimulation blocks endothelial cell activation and leukocyte recruitment during inflammation J. Exp. Med 201 7 1113 23 15809354 10.1084/jem.20040463 PMC2213139

[b75-ccrpm-2008-035] Shanahan F 1999 Brain-gut axis and mucosal immunity: a perspective on mucosal psychoneuroimmunology Semin. Gastrointest Dis 10 1 8 13 10065767

[b76-ccrpm-2008-035] Shepherd AJ Downing JE Miyan JA 2005 Without nerves, immunology remains incomplete -in vivo veritas Immunology 116 2 145 63 16162264 10.1111/j.1365-2567.2005.02223.x PMC1817817

[b77-ccrpm-2008-035] Shishehbor MH Alves C Rajagopal V 2007 Inflammation: implications for understanding the heart-brain connection Cleve. Clin. J. Med 74 Suppl 1 S37 S41 17455542 10.3949/ccjm.74.suppl_1.s37

[b78-ccrpm-2008-035] Siler TM Swierkosz JE Hyers TM Fowler AA Webster RO 1989 Immunoreactive interleukin-1 in bronchoalveolar lavage fluid of high-risk patients and patients with the adult respiratory distress syndrome Exp. Lung Res 15 6 881 94 2612445 10.3109/01902148909069633

[b79-ccrpm-2008-035] Solway J Leff AR 1991 Sensory neuropeptides and airway function J. Appl. Physiol 71 6 2077 87 1663932 10.1152/jappl.1991.71.6.2077

[b80-ccrpm-2008-035] Sonea IM Palmer MV Akili D Harp JA 2002 Treatment with neurokinin-1 receptor antagonist reduces severity of inflammatory bowel disease induced by Cryptosporidium parvum Clin. Diagn. Lab Immunol 9 2 333 40 11874873 10.1128/CDLI.9.2.333-340.2002 PMC119952

[b81-ccrpm-2008-035] Sorkin LS Xiao WH Wagner R Myers RR 1997 Tumour necrosis factor-alpha induces ectopic activity in nociceptive primary afferent fibres Neuroscience 81 1 255 62 9300418 10.1016/s0306-4522(97)00147-4

[b82-ccrpm-2008-035] Soukhova G Wang Y Ahmed M Walker JF Yu J 2003 Bradykinin stimulates respiratory drive by activating pulmonary sympathetic afferents in the rabbit J. Appl. Physiol 95 1 241 9 12679361 10.1152/japplphysiol.00582.2002

[b83-ccrpm-2008-035] Steinsson K arcon-Riquelme ME 2005 Genetic aspects of rheumatic diseases Scand. J. Rheumatol 34 3 167 77 16134722 10.1080/03009740510026779

[b84-ccrpm-2008-035] Straub RH Cutolo M 2001 Involvement of the hypothalamic—pituitary—adrenal/gonadal axis and the peripheral nervous system in rheumatoid arthritis: viewpoint based on a systemic pathogenetic role Arthritis Rheum 44 3 493 507 11263762 10.1002/1529-0131(200103)44:3<493::AID-ANR95>3.0.CO;2-U

[b85-ccrpm-2008-035] Suter PM Suter S Girardin E Roux-Lombard P Grau GE Dayer JM 1992 High bronchoalveolar levels of tumor necrosis factor and its inhibitors, interleukin-1, interferon, and elastase, in patients with adult respiratory distress syndrome after trauma, shock, or sepsis Am. Rev. Respir. Dis 145 5 1016 22 1586041 10.1164/ajrccm/145.5.1016

[b86-ccrpm-2008-035] Tracey KJ 2002 The inflammatory reflex Nature 420 6917 853 9 12490958 10.1038/nature01321

[b87-ccrpm-2008-035] van Westerloo DJ Giebelen IA Florquin S Bruno MJ Larosa GJ Ulloa L Tracey KJ van der PT 2006 The vagus nerve and nicotinic receptors modulate experimental pancreatitis severity in mice Gastroenterology 130 6 1822 30 16697744 10.1053/j.gastro.2006.02.022

[b88-ccrpm-2008-035] Vassilopoulos D Mantzoukis D 2006 Dialogue between the brain and the immune system in inflammatory arthritis Ann. N.Y. Acad. Sci 1088 132 8 17192561 10.1196/annals.1366.031

[b89-ccrpm-2008-035] von LA Ehnes F Dahme B 2006 Emotions and respiratory function in asthma: a comparison of findings in everyday life and laboratory Br. J. Health Psychol 11 Pt 2 185 98 16643693 10.1348/135910705X52462

[b90-ccrpm-2008-035] Wagner R Myers RR 1996 Schwann cells produce tumor necrosis factor alpha: expression in injured and non-injured nerves Neuroscience 73 3 625 9 8809782 10.1016/0306-4522(96)00127-3

[b91-ccrpm-2008-035] Wald A 2002 Psychotropic agents in irritable bowel syndrome J. Clin. Gastroenterol 35 1 S53 S57 12184140 10.1097/00004836-200207001-00010

[b92-ccrpm-2008-035] Wang H Liao H Ochani M Justiniani M Lin X Yang L Al-Abed Y Wang H Metz C Miller EJ Tracey KJ Ulloa L 2004 Cholinergic agonists inhibit HMGB.1 release and improve survival in experimental sepsis Nat. Med 10 11 1216 21 15502843 10.1038/nm1124

[b93-ccrpm-2008-035] Wang H Yu M Ochani M Amella CA Tanovic M Susarla S Li JH Wang H Yang H Ulloa L Al-Abed Y Czura CJ Tracey KJ 2003a Nicotinic acetylcholine receptor alpha7 subunit is an essential regulator of inflammation Nature 421 6921 384 8 12508119 10.1038/nature01339

[b94-ccrpm-2008-035] Wang X Wang BR Duan XL Zhang P Ding YQ Jia Y Jiao XY Ju G 2002 Strong expression of interleukin-1 receptor type I in the rat carotid body J. Histochem. Cytochem 50 12 1677 84 12486091 10.1177/002215540205001213

[b95-ccrpm-2008-035] Wang Y Soukhova G Proctor M Walker J Yu J 2003b Bradykinin causes hypotension by activating pulmonary sympathetic afferents in the rabbit J. Appl. Physiol 95 1 233 40 12679362 10.1152/japplphysiol.00584.2002

[b96-ccrpm-2008-035] Watkins LR Goehler LE Relton JK Tartaglia N Silbert L Martin D Maier SF 1995 Blockade of interleukin-1 induced hyperthermia by subdiaphragmatic vagotomy: evidence for vagal mediation of immune-brain communication. *Neurosci.* Lett 183 1–2 27 31 10.1016/0304-3940(94)11105-r 7746479

[b97-ccrpm-2008-035] Webster JI Tonelli L Sternberg EM 2002 Neuroendocrine regulation of immunity Annu. Rev. Immunol 20 125 63 11861600 10.1146/annurev.immunol.20.082401.104914

[b98-ccrpm-2008-035] Yu J Lin S Zhang J Otmishi P Guardiola JJ 2007 Airway nociceptors activated by pro-inflammatory cytokines Respir. Physiol. Neurobiol 156 116 9 17182290 10.1016/j.resp.2006.11.005

[b99-ccrpm-2008-035] Zelenka M Schafers M Sommer C 2005 Intraneural injection of interleukin-1beta and tumor necrosis factor-alpha into rat sciatic nerve at physiological doses induces signs of neuropathic pain Pain 116 3 257 63 15964142 10.1016/j.pain.2005.04.018

